# Lung transcriptional unresponsiveness and loss of early influenza virus control in infected neonates is prevented by intranasal *Lactobacillus rhamnosus* GG

**DOI:** 10.1371/journal.ppat.1008072

**Published:** 2019-10-11

**Authors:** Ogan K. Kumova, Adam J. Fike, Jillian L. Thayer, Linda T. Nguyen, Joshua Chang Mell, Judy Pascasio, Christopher Stairiker, Leticia G. Leon, Peter D. Katsikis, Alison J. Carey

**Affiliations:** 1 Microbiology and Immunology, Drexel University College of Medicine, Philadelphia, PA, United States of America; 2 Pediatrics, Drexel University College of Medicine, Philadelphia, PA, United States of America; 3 Pathology, Drexel University College of Medicine, Philadelphia, PA, United States of America; 4 Immunology, Erasmus University Medical Center, Rotterdam, the Netherlands; Johns Hopkins Bloomberg School of Public Health, UNITED STATES

## Abstract

Respiratory viral infections contribute substantially to global infant losses and disproportionately affect preterm neonates. Using our previously established neonatal murine model of influenza infection, we demonstrate that three-day old mice are exceptionally sensitive to influenza virus infection and exhibit high mortality and viral load. Intranasal pre- and post-treatment of neonatal mice with *Lactobacillus rhamnosus* GG (LGG), an immune modulator in respiratory viral infection of adult mice and human preterm neonates, considerably improves neonatal mice survival after influenza virus infection. We determine that both live and heat-killed intranasal LGG are equally efficacious in protection of neonates. Early in influenza infection, neonatal transcriptional responses in the lung are delayed compared to adults. These responses increase by 24 hours post-infection, demonstrating a delay in the kinetics of the neonatal anti-viral response. LGG pretreatment improves immune gene transcriptional responses during early infection and specifically upregulates type I IFN pathways. This is critical for protection, as neonatal mice intranasally pre-treated with IFNβ before influenza virus infection are also protected. Using transgenic mice, we demonstrate that the protective effect of LGG is mediated through a MyD88-dependent mechanism, specifically via TLR4. LGG can improve both early control of virus and transcriptional responsiveness and could serve as a simple and safe intervention to protect neonates.

## Author summary

Viral lung infections are the number one reason for hospitalization of children under the age of 5 and are a major public health concern. Premature babies, or those born before 37 weeks gestation, are particularly susceptible to viral infections, but exact mechanisms for this susceptibility have not been determined. Here, using a pre-clinical infant mouse model of influenza virus infection, we have found increased neonatal susceptibility to respiratory viral infection compared to adults, and we demonstrate that a probiotic administered intranasally prior to infection provides dramatic protection to infant mice by inducing production of a key anti-viral cytokine, type I interferons.

## Introduction

Respiratory infection in preterm and term neonates is a major public health problem. Respiratory syncytial virus (RSV) bronchiolitis is the leading cause of infant hospitalization in the United States annually [1], and globally, influenza virus causes approximately 374,000 respiratory hospitalizations per year in children <1 y of age (including 270,000 among those less than 6 months) [2]. The most significant risk factors for hospitalization due to an acute lower respiratory tract infection are prematurity and age [3]. Late preterm infants have significantly higher risk for respiratory disease and infections, which contributes to the use of twice as many healthcare dollars over the first 2 years of life, as compared to their term counterparts [4].

During this critical window of susceptibility to viral infection, neonates begin to be colonized with a variety of microbiota which are molded into niche-specific bacterial communities [5, 6]. These commensal communities are highly dynamic during the first few months of life for preterm and term neonates and are impacted by external factors, such as antibiotic exposure, mode of neonatal delivery and diet [7]. Differences in the dominant airway microbial communities in these first few months of life has been linked to susceptibility to respiratory infections [8]. In addition, commensal-derived signals establish an activation threshold of the innate immune system required for optimal antiviral immunity [9]. Therefore, modulating early airway microbial communities presents a potential therapeutic strategy to prevent or ameliorate respiratory tract infections. Indeed, local treatment in the form of intranasal administration of *Lactobacillus rhamnosus* GG (LGG) [10] and *Lactobacillus casei* [11, 12] to adult mice significantly reduces the symptoms and increased survival rates in influenza virus-infected mice.

Clinical epidemiologic observations further suggest that the immune effects of early-life microbial exposure persist into later life [13]. Similarly, the murine microbiome can influence anti-viral immunity against influenza infection [14]. The use of multiple doses of intranasal probiotics augments the immune response of adult mice infected with influenza virus and RSV, demonstrating their protective role in the context of viral infections [15–19]. Clinical studies suggest that oral LGG might protect children from influenza virus infection through an interaction with gut-associated immune tissue by indirectly up-regulating respiratory immunity [20–22].

To date, investigation of probiotic treatments to protect the host from respiratory infections have used oral administration, which presumably acts systemically [15–19]. We hypothesized that intranasal LGG would provide neonatal mice with protection from the sequelae of influenza virus infection. To test this hypothesis, we used our previously established murine model of neonatal influenza virus infection [23] to investigate neonatal susceptibility to respiratory viral infection and determine whether intranasal LGG treatment prior to infection provides protection. Probiotics may accelerate antiviral protection by stimulating the production of type I Interferons (IFNs) [12, 24, 25]. We demonstrate here that 3-day old mice are exceptionally susceptible to influenza virus infection and have minimal transcriptional responses to the infection at 12 hours post-infection, which begins to improve by 24 hours post-infection, demonstrating a delay in the kinetics of the neonatal antiviral response. Administration of LGG prior to influenza infection dramatically improves survival and provides an early increase in transcription of type I IFNs. This LGG-mediated protection is MyD88-dependent and specifically involves TLR4 recognition of LGG.

## Results

### Neonatal mice are highly susceptible to influenza virus

**Fig 1:**
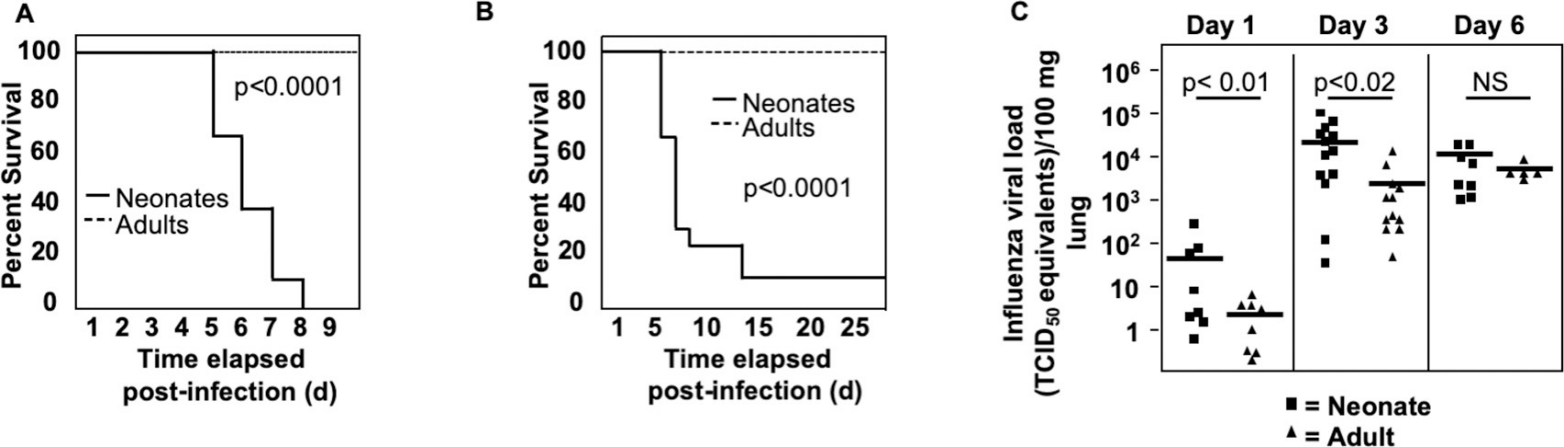
Decreased survival of influenza virus-infected neonatal mice coupled with loss of early viral control. 3-day old neonatal and 8 week-old adult mice were intranasally infected with PR8 influenza virus. **(A)** Neonates were infected with the same sublethal adult virus dose. Neonatal mice (n = 8) and adult mice (n = 4) from 2 independent experiments. **(B)** Neonates were infected with one fifth of a sublethal adult virus dose. Neonatal mice (n = 24) and adult mice (n = 6) from 3 independent experiments. **(C)** 3-day old neonatal and 8 week-old adults mice were intranasally infected with the same dose of influenza virus as **(B)**. Mice were harvested at 1, 3, and 6 days post-infection shown. Viral loads in lungs were measured by RT-PCR.

We have previously described a reduced and delayed primary CD8^+^ T cell response to influenza virus in 3-day old neonatal mice [23]. Using our established neonatal infection model, 3-day old and 8-week old adult C57BL/6 mice were infected with H1N1 influenza virus strain A/Puerto Rico/8/1934 (PR8) and followed for morbidity and mortality. Three-day old mice were exquisitely sensitive to influenza virus infection and exhibited high mortality. When 3-day old mice were infected with the same weight-adjusted dosage of PR8 virus (0.15 TCID_50_ /g weight) as adults, 100% succumbed within 8 days (Fig 1A). When neonatal mice were infected with a four-fold lower dose of PR8 influenza virus than their adult counterparts (0.04 TCID_50_ /g versus 0.15 TCID_50_/g, for neonate and adult mice respectively), only 20% of neonates survived by 7 days post-infection. Ultimately, only 10% of neonates survived this low-dose challenge, while 100% of adults survived a standard dose challenge (Fig 1B). Despite the much lower infecting dose, neonatal mice have profoundly increased mortality, particularly between 5 and 8 days post-infection compared to adult mice.

With this early mortality, we asked whether there were differences in viral load early in infection. Neonatal animals were infected and harvested at 1, 3, and 6 days post-infection. Viral loads were significantly higher in neonatal animals as compared to adult animals at 1 day (p<0.01) and 3 days post-infection (p<0.02) (Fig 1C), but were similar to adult animals by 6 days post-infection, suggesting that neonates fail to respond to viral infection during early stages of infection.

### Treatment with LGG prior to influenza infection protects neonatal mice

**Fig 2:**
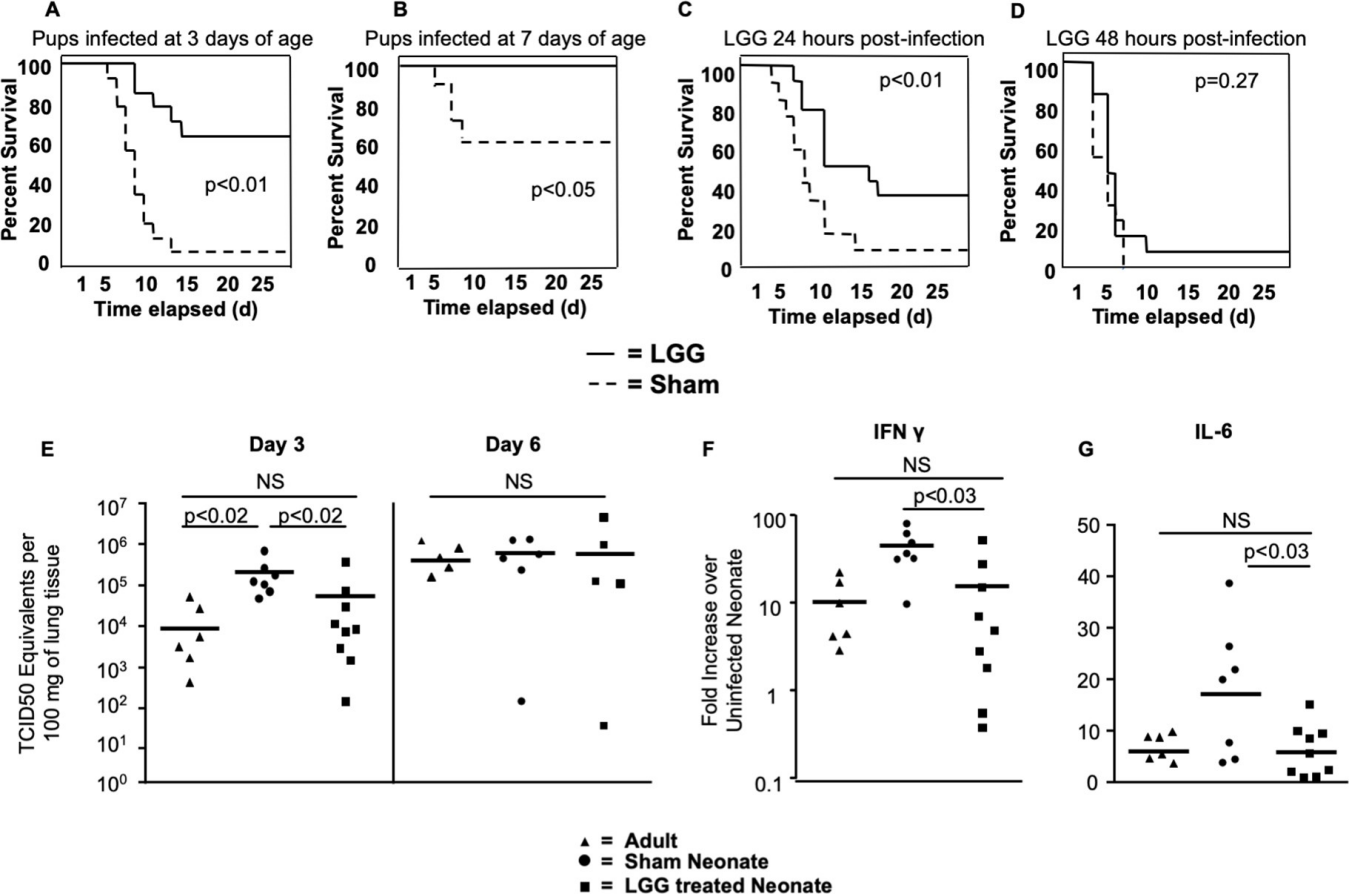
Improved survival and decreased viral loads of neonatal mice when pretreated with *Lactobacillus* prior to Influenza infection. **(A)** Mouse pups received 1 x10^6^ Colony Forming Units of LGG intranasally on Days 1 and 2 of life. On Day 3, pups were infected intranasally with PR-8 influenza, and survival was monitored. n = 15 per group from 3 independent experiments. **(B)** To test if LGG protected older infant mice, mouse pups were given 1.5 x10^6^ Colony Forming Units of LGG intranasally on Day 6 of life. On Day 7, pups were infected intranasally with PR8 influenza, and survival was monitored. n = 14 per group from 3 independent experiments. To test if LGG conferred protection after influenza infection, mice were infected on the third day of life with influenza. **(C)** 24 hours or **(D)** 48 hours later, mice were given 1 x10^6^ Colony Forming Units of LGG intranasally. n = 16 per group from 4 independent experiments. **(E)** Viral loads were determined at 3 and 6 days post infection by real time PCR. Data from 3 independent experiments. Real time PCR was performed to determine transcription of **(F)** IFNγ and **(G)** IL-6 at 3 days post-infection. Data from 4 independent experiments.

*Lactobacillus* species have shown promise as immune adjuvants in viral infections in adult animal models [14, 26, 27]. Based on our observation of limited and delayed neonatal responses to viral infection (Fig 1B and 1C), we asked whether probiotic administration of LGG could protect neonatal mice. On Days 1 and 2 of life, neonatal mice were given 2 separate intranasal doses of 1x10^6^ colony forming units (CFU) of live LGG or similarly diluted Lactobacilli MRS growth media as a sham control (Sham). Both groups of mice were then infected with the same dose (0.04TCID_50_ /g) of PR8 influenza virus on Day 3 of life. We chose to treat mice intranasally with LGG to directly apply LGG to the respiratory mucosa. Furthermore, we chose a brief, two-day course for the practical reason that the mouse immune system rapidly changes in the first week of life [23], compared to adult murine studies where probiotics are given over several weeks [26, 28]. We found that intranasal administration of LGG on the first and second day of life prior to influenza infection profoundly improved survival, with 65% of the pups surviving who received the probiotic (LGG), as compared to only 10% survival of pups receiving sham treatment (Sham) (p<0.01) (Fig 2A).

Others have found that intranasal application of LGG prior to influenza infection in adult mice is protective, but this requires a multi-dose protocol over a 3–4 week period, using 10–100 times more CFU per dose than our treatment [10, 29]. Potentially, slightly older neonatal mice may not have the additional benefit from a single dose of LGG, compared to 3-day old mice, which have minimal respiratory tract microbial colonization. To test this, we pretreated 6-day old neonatal mice and infected the animals at Day 7 of life with a 0.04 TCID_50_/g dose. Although mice at this age were not as sensitive to influenza as 3-day old mice, a single dose (1.5 million CFU) of live LGG given at Day 6 of life and 1 day before influenza virus infection was still protective (p<0.05) (Fig 2B). These results demonstrate that sensitivity to influenza virus infection is age-dependent in neonatal mice with ~10–20% survival of 3-day old mice increasing to ~60% for 7-day old mice and 100% for adult mice. These results also reveal that intranasal LGG administration clearly provides protection to neonatal mice from a respiratory infection and this protection is still afforded at 1 week of age in this infection model.

### Timing of administration of intranasal LGG is critical for protection

Next, we asked whether LGG administration *after* influenza infection could confer similar degrees of protection. Animals were infected on Day 3 of life and then received a dose of live LGG 24 hours (Fig 2C) or 48 hours (Fig 2D) after infection. When LGG was administered 24 hours after infection, there was a statistically significant improvement in survival between those animals receiving the LGG and those receiving sham treatment (p<0.01). Although the difference in survival was smaller than when LGG was administered prior to infection, it still conferred a 4.5-fold increase in survival. When animals received LGG 48 hours post-infection, protective benefits of LGG treatment were lost (p = 0.27), indicating a narrow time window within the post-infection period for LGG to be effective.

### Neonates pre-treated with LGG have low influenza viral loads and low levels of pro-inflammatory cytokines

To further understand the mechanism by which LGG protects neonates, we asked whether LGG treatment reduced viral loads, since neonatal mice exhibited impaired viral control compared to adult mice (Fig 1C). We infected LGG-treated 3-day old neonatal pups with PR8 influenza virus and compared their lung viral loads to sham-treated 3-day old neonatal pups and adult mice. In agreement with our results above, at 3 days post-infection (DPI), sham-treated 3-day old neonatal pups had significantly higher viral loads than sham-treated adult mice (p<0.02) (Fig 2E). Furthermore, LGG-treated pups had lower viral loads compared to sham-treated pups (p<0.02), comparable to viral loads in adult controls (Fig 2E). As shown above (Fig 1C), by 6 DPI no differences in viral load were found between the LGG-treated mice and the sham-treated pups and adults. Therefore, neonatal pups that received LGG had better control of viral infection at early time points. We next asked whether LGG-treated neonatal animals had less induction of pro-inflammatory cytokines because of the decreased early viral loads. We performed real-time PCR on whole lungs to determine transcription levels of both *IFNG* (Fig 2F) and *IL6* (Fig 2G) and found these levels to be reduced in neonatal pups treated with LGG compared to sham-treated pups (p<0.03).

### Neonatal pups pretreated with LGG have similar pathology severity scores

**Fig 3:**
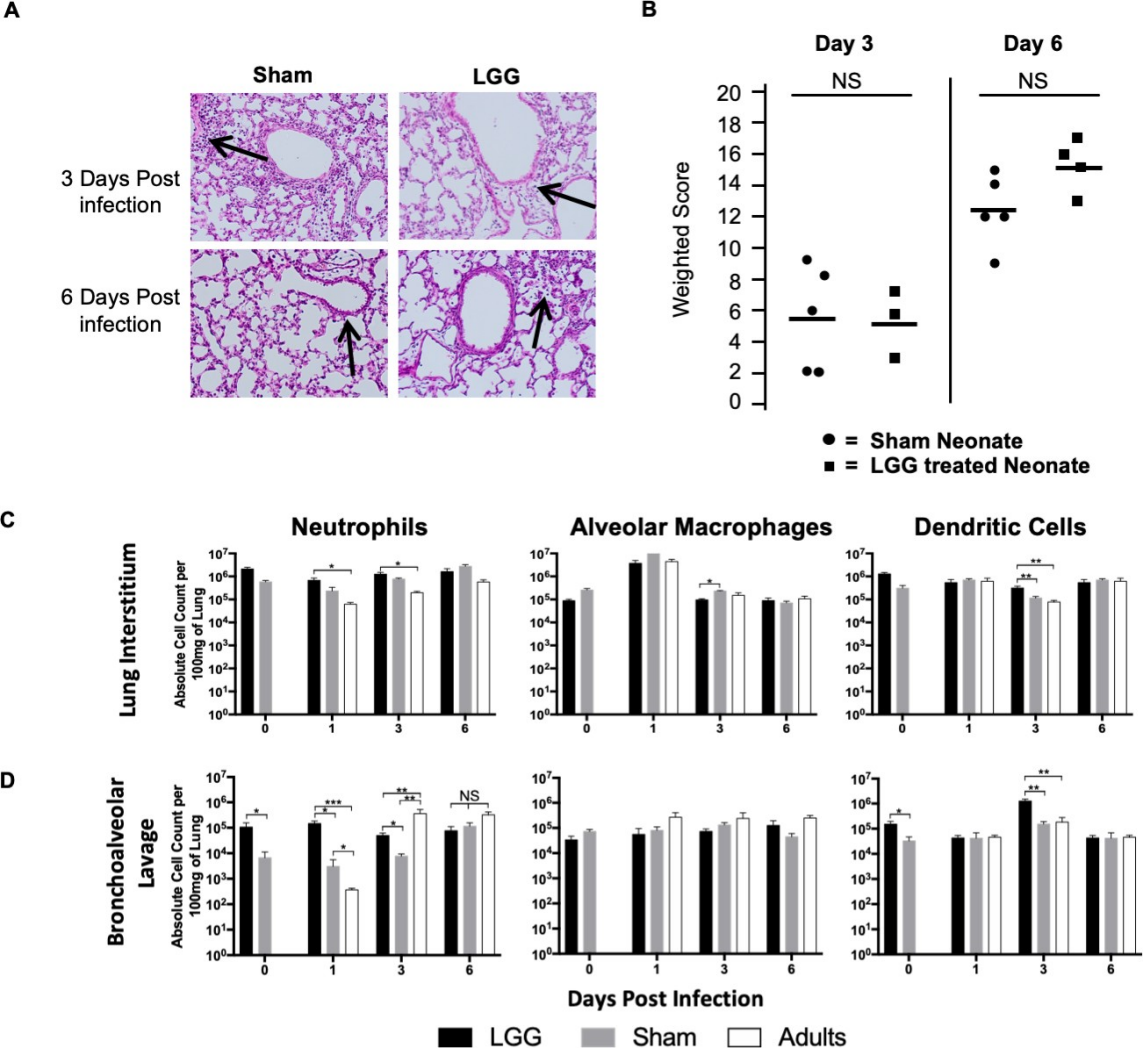
Differential recruitment of immune cells to the alveolar airspace in neonatal animals. Mouse pups received 1 x10^6^ Colony Forming Units of LGG intranasally on Days 1 and 2 of life. On Day 3, pups were infected intranasally with PR-8 influenza. **(A)** Histopathology was done at 3 and 6 days post-infection, which demonstrated similar bronchopulmonary infiltrates in both sham and LGG treated neonates. H&E Stain, representative figures. Arrows indicate areas of bronchopulmonary infiltration. Clinical severity scoring was performed **(B)**. In separate experiments, cell counts for the specified cell types from **(C)** whole lung tissue and **(D)** bronchoalveolar lavage was determined by flow cytometry. Data from 3 independent experiments.

In addition to massively improved survival of pups pre-treated with LGG, mortality was also delayed (Fig 2A). Therefore, we asked whether lung pathology differed early in infection with LGG pre-treatment, which could contribute to improved survival in the first week of infection. Lung histopathology from pups pretreated with LGG was performed and compared to sham-treated pups. A weighted scoring system was used [30], which accounts for both the intensity of pathology and the percentage of lung affected. Animals were scored on the severity of alveolitis and peribronchiolitis and the percentage of affected lung in each of these respective categories by a pathologist blinded to the treatment groups. At 3 and 6 DPI, neonates pre-treated with LGG had similar broncho-pneumonia and clinical severity scores, compared to neonatal animals given the sham treatment (Fig 3A and 3B). Although surprising, pathomorphological features do not always reflect disease severity or outcome during an early influenza infection [31].

Next, we asked whether LGG pre-treatment resulted in differential recruitment of important innate immune cell populations into the airway and the site of infection. Therefore, we pretreated pups with LGG and infected with influenza virus and harvested at 1, 3 and 6 DPI. To determine the impact of LGG alone on immune cell recruitment, we also treated with LGG and harvested the animals at 3-days of life, the time of influenza virus infection. In whole lung, there were no significant differences in the alveolar macrophages (CD11c^+^SiglecF^+^) or the recruitment of neutrophils (CD11b^+^Ly6G and dendritic cells (CD11c^+^MHC^Hi^) between LGG- and sham-treated animals (Fig 3C), which is consistent with the pathology results (Fig 3A). However, we did observe significant differences in the infiltration of neutrophils and dendritic cells into the airways, as determined by bronchoalveolar lavage, consistent with previous results[32]. No changes in alveolar macrophages were detected. (Fig 3D). Recruitment of neutrophils increased 10-fold with LGG alone in the absence of influenza infection at 0 days post-infection (p<0.05), but strikingly, no change was observed in sham-treated infected animals at 1 or 3 days post-infection compared to uninfected animals (Day 0) (Fig 3C and 3D). Recruitment of neutrophils to the alveolar airspace is correlated with a favorable outcome in mild and severe influenza A strains including PR8[33]. In contrast, adults have increased neutrophil recruitment to the alveolar airspace between 1 and 3 days of infection. LGG-treated animals show an intermediate recruitment of neutrophils (Fig 3D). All together, these results highlight how LGG pre-treatment provides an adult-like immune phenotype to neonates.

### LGG-treated neonates exhibit an adult-like transcriptional signature in the lungs compared to sham-treated counterparts

**Fig 4:**
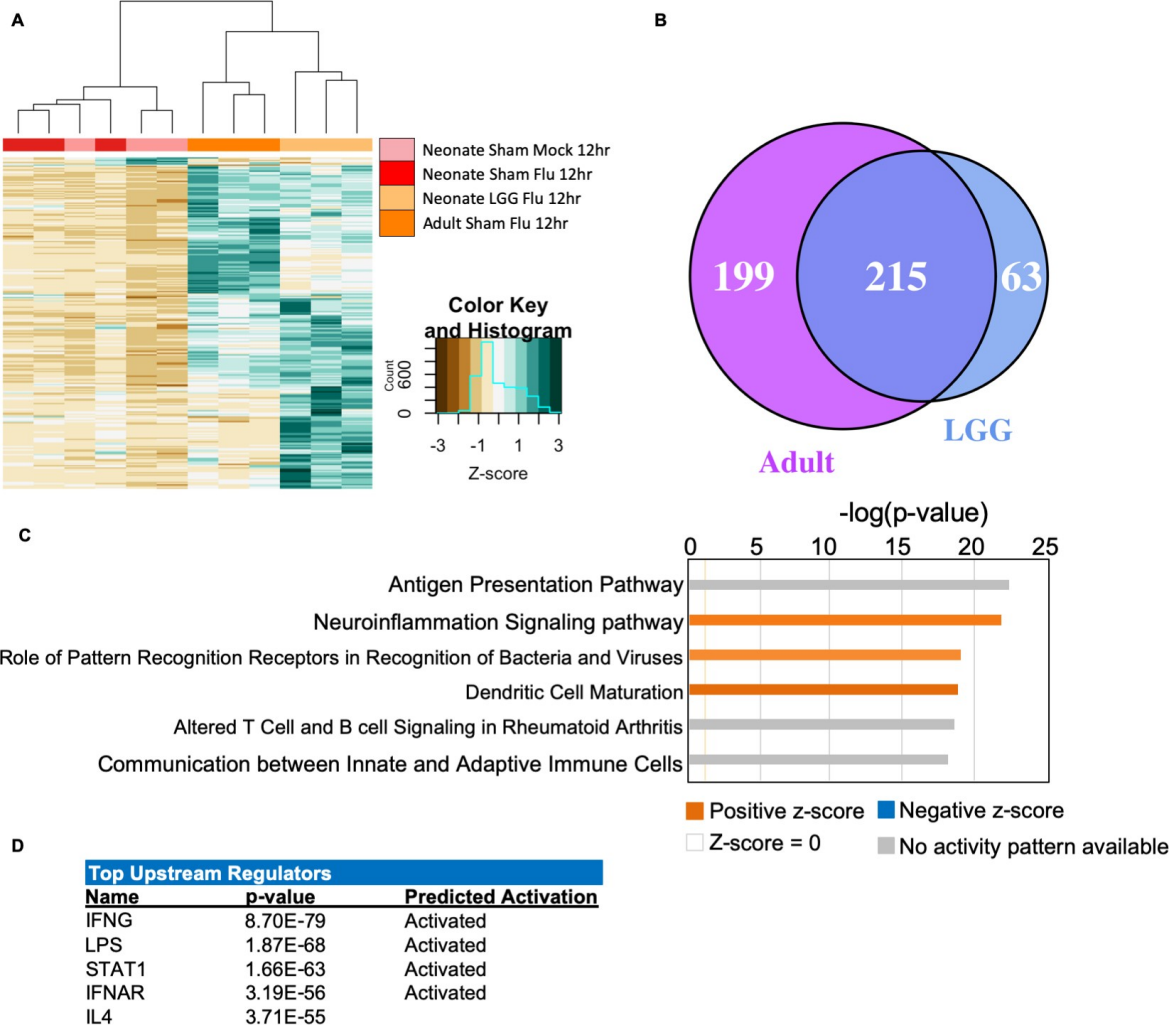
*Lactobacillus* causes differential transcriptional response to influenza. Neonatal animals were given either LGG or sham treatment, and then infected with influenza on the third day of life. Animals were harvested 12 hours post-infection. Nanostring gene transcription analysis was performed on lungs. **(A)** Heatmap of all genes test in the LGG treated neonates, compared to expression in the other groups. Green: Upregulated; Brown: Down regulated. N = 3–4 per group, 2 experiments. **(B)** Venn diagram depicting the overlap of differentially regulated genes in the adult and the LGG-treated neonate. **(C)** IPA analysis of canonical pathways upregulated in the LGG-treated neonates. Significance P-values were calculated based on the Fisher's right tailed exact test. The log (p-value) are shown on the x-axis of the bar chart. The color of the bars indicates the activity (orange bars) or the inhibition (blue bars) of the predicted pathways. **(D)** IPA Top upstream regulators of gene expression in LGG-treated neonates versus uninfected neonates.

We next investigated how LGG treatment prior to influenza virus infection mediated early immune responses in neonates. We measured RNA transcriptional changes in 753 immune-related genes by Nanostring technology in neonatal (LGG- and sham-treated), and adult whole lungs 12 hours after PR8 influenza virus infection, and uninfected age-matched neonates. These four experimental groups were normalized to their own housekeeping genes. Notably, transcriptional signatures of sham-treated infected neonates clustered with those of uninfected neonates, while the LGG-treated influenza infected neonates were more similar to adults, with both groups showing strong induction of many immune-related genes (Fig 4A). We next defined sets of differentially expressed genes in the three experimental groups (Sham, LGG, and Adult) compared to untreated uninfected controls (Uninfected). Sham-treated neonates infected with influenza virus had only 16 differentially expressed genes in the lungs, compared to uninfected neonates. In contrast, adult mice had 414 differentially expressed genes compared to uninfected neonates of the 753 tested (55%). Similarly, LGG-treated influenza infected neonatal lungs had 278 differentially expressed genes of the 753 tested (37%) compared to uninfected neonates; 194 of these genes were induced by more than 2-fold (S1 Table). Therefore, the transcriptional unresponsiveness to influenza virus infection in neonatal lungs was improved by LGG pretreatment. Indeed, of the 278 differentially expressed genes in LGG-treated neonates, 215 were also induced in adults (Fig 4B, S2 Table), whereas sham-treated infected neonates at 12 hours post-infection had highly similar transcriptional profiles to uninfected neonatal animals.

To define the key pathways induced in LGG-treated neonates, we applied canonical pathway analysis with Ingenuity Pathway Analysis (IPA) based on the 215 genes defined above (Fig 4C). The top canonical pathways included several related to innate pathogen recognition, notably two: “Role of Pattern Recognition Receptors in Recognition of Bacteria and Viruses” and “Dendritic Cell Maturation”. The top upstream regulators defined by this analysis were IFNG, LPS, STAT1, IFNAR and IL4 (Fig 4D). These transcriptional changes indicate that neonatal mice have a reduced early transcriptional response to influenza virus infection. Furthermore, LGG pre-treatment primed neonates to respond to influenza virus infection more like adults and primarily increased early IFN responses at 12 hours post-infection.

### Neonates have a delayed recognition and response to influenza virus infection

**Fig 5:**
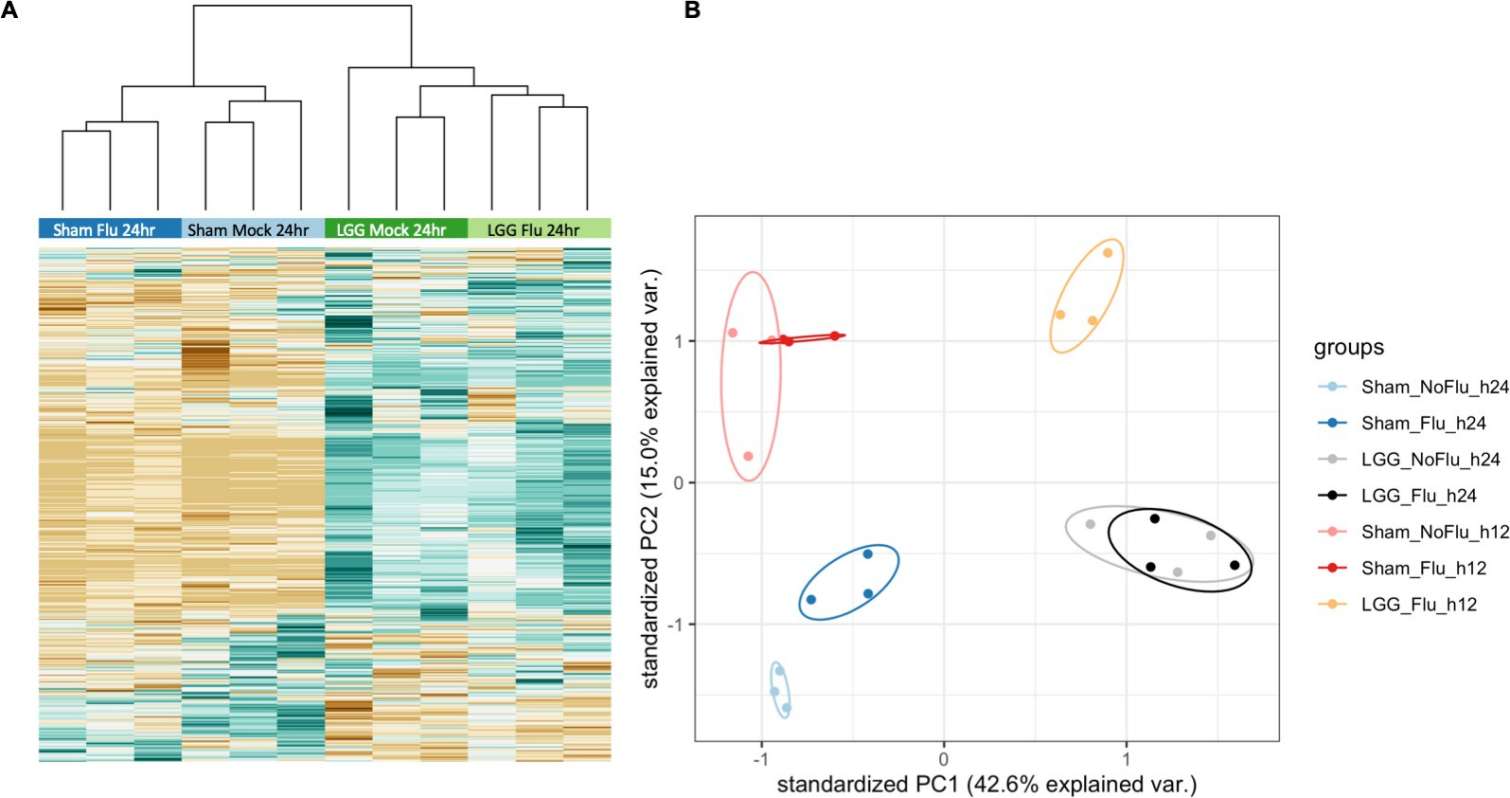
Sham-treated neonates begin to respond 24 hours post-infection. LGG or sham treated animals were either mock or influenza infected on Day 3 of life. Animals were harvested 24 hours post-infection. Nanostring gene transcription analysis was performed on whole lungs. **(A)** Heatmap of all genes assayed in the LGG-treated neonates, compared to expression in all other groups. Green: Up regulated; Brown: Down regulated. N = 3 per group. **(B)** Principal component analysis of both 12 and 24 hours post infection samples.

Given the neonatal transcriptional paralysis to influenza infection at 12 hours post-infection, we asked whether this paralysis persisted later in infection by comparing transcriptional changes in neonatal whole lungs 24 hours after PR8 influenza virus infection to age-matched uninfected neonates. To determine the impact of LGG pre-treatment alone on transcription, we also measured transcriptional induction of immune-related genes by including LGG-treated, uninfected neonates and LGG-treated, infected neonates at 24 hours. Similar to the 12-hour time point, sham-treated and uninfected neonates freely clustered, as did LGG-infected neonates and LGG-uninfected neonates (Fig 5A). Principal component analysis of both 12-hour and 24-hour time points also indicated little variation between the uninfected and infected neonates at both time points, which potentially indicates a developmentally-regulated transcriptional response (Fig 5B). There was also little variation among the animals which received the LGG alone and those animals which received LGG and were infected with influenza virus. Therefore, most of the immune-related transcriptional changes in the LGG-treated infected neonates was driven by LGG and not the infection. Adults distinctly separated away from the neonates (S1 Fig), which again supports a developmental regulation.

### LGG treatment alone is responsible for induction of inflammatory genes

**Fig 6:**
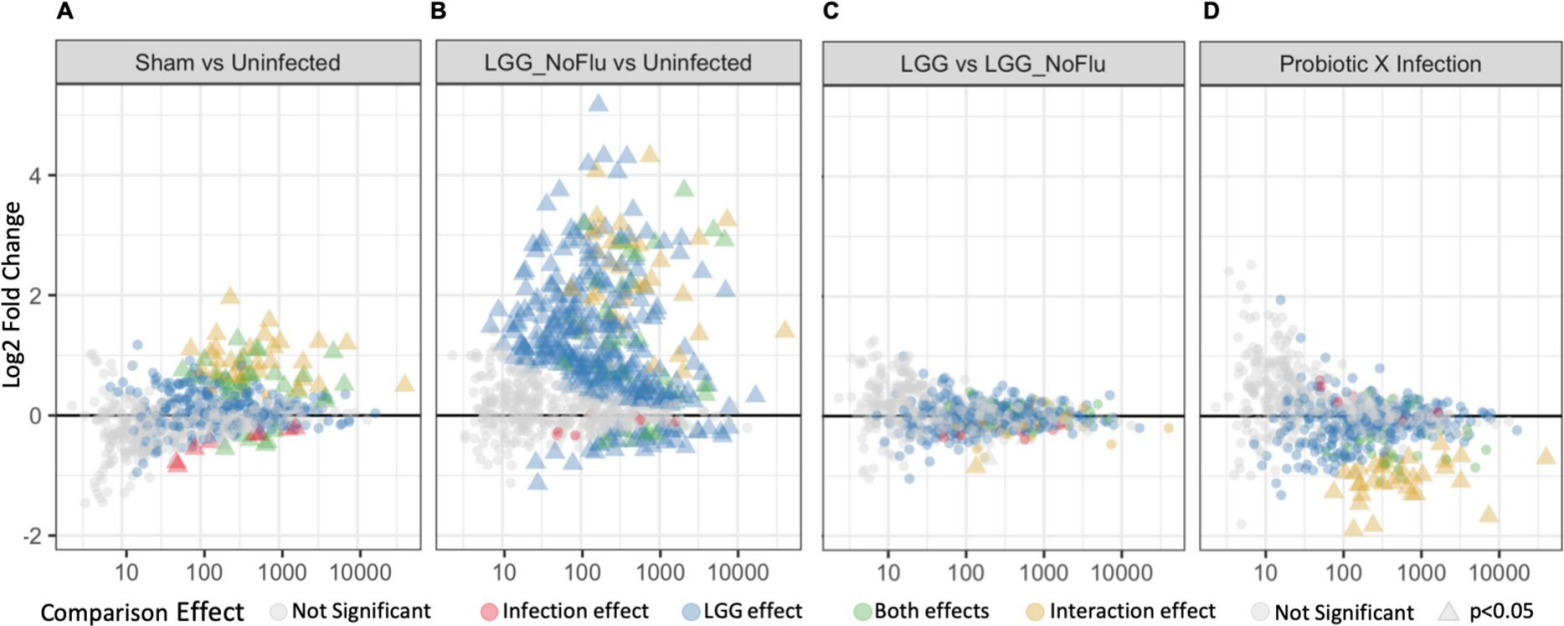
LGG treatment alone is responsible for induction of inflammatory genes. Neonatal animals were given either LGG or sham treatment, and then infected with influenza or sham on the Day 3 of life. Animals were harvested 24 hours post-infection. Nanostring gene transcription analysis was performed on lungs. MA plot of all genes tested at 24 hours post-infection, with **(A)** sham-treated influenza infected neonates compared to uninfected neonates; **(B)** LGG-treated uninfected neonates compared to uninfected neonates; **(C)** LGG-treated infected neonates compared to LGG-treated uninfected neonates; and **(D)** LGG-treated infected neonates compared to sham-treated infected neonates. Triangles denote statistical significance (p<0.05). Red: genes unique to infections; blue: genes unique to probiotic treatment; green: genes that are expressed by both probiotic and influenza infected animals *independently*; gold: interaction effect of LGG and influenza.

Next, we sought to identify differentially expressed genes due to influenza virus infection or LGG treatment alone compared to uninfected neonates at 24 hours. More immune-related genes were induced upon influenza virus infection by 24 hours compared to 12 hours (75 and 16 significant differentially expressed genes, respectively, (Fig 6A), which demonstrates that the neonatal animal has begun to upregulate important inflammatory pathways. Of these, 8 genes were only differentially expressed in infection without probiotic treatment (red triangles), whereas 67 genes were also differentially expressed with probiotic treatment alone (green and gold triangles). When neonates which receive LGG without influenza virus infection were compared to uninfected neonatal animals, 357 genes showed significant differential expression (Fig 6B). Of these, 290 genes were specifically induced by the probiotic (blue triangles, along with the 67 induced by both viral infection and probiotic treatment alone (green and gold triangles). In sharp contrast, only 3 genes were differentially expressed between LGG treatment alone and virally infected neonates pre-treated with LGG (Fig 6C), notably the downregulated gene, CAMP, encoding Catheliciden Antimicrobial Peptide, which plays a role in antimicrobial defenses and leads to activation of an inflammatory response[34].

Finally, genes were identified showing a significant interaction effect between influenza infection and LGG treatment, whereby the contributions of infection or treatment alone do not additively predict their combination (Fig 6D). There were 32 genes upregulated by influenza infection alone (Fig 6A, gold triangles) and LGG treatment alone (Fig 6B, gold triangles). However, neonates pretreated with LGG prior to infection had dampened induction of these 32 genes, which accounted for all of the significant interaction effects (Fig 6D, gold triangles). The 32 genes with their associated functions are in S3 Table. Canonical pathway analysis was completed with Ingenuity Pathway Analysis (IPA) based on these 32 genes and the 72 differentially expressed genes in sham-infected neonates relative to uninfected age-matched neonates at 24 hours post-infection. This analysis revealed one pathway which was down-regulated in LGG-treated infected neonates and upregulated in sham-treated infected neonates: “Nitric oxide and Reactive Oxygen Species in Macrophages”. Taken together, these results demonstrate the neonatal inability to respond to influenza during early infection in the absence of LGG treatment. LGG restores an adult-like transcriptome to neonates in this infection model.

### LGG promotes early transcription of Type I IFNs

**Fig 7:**
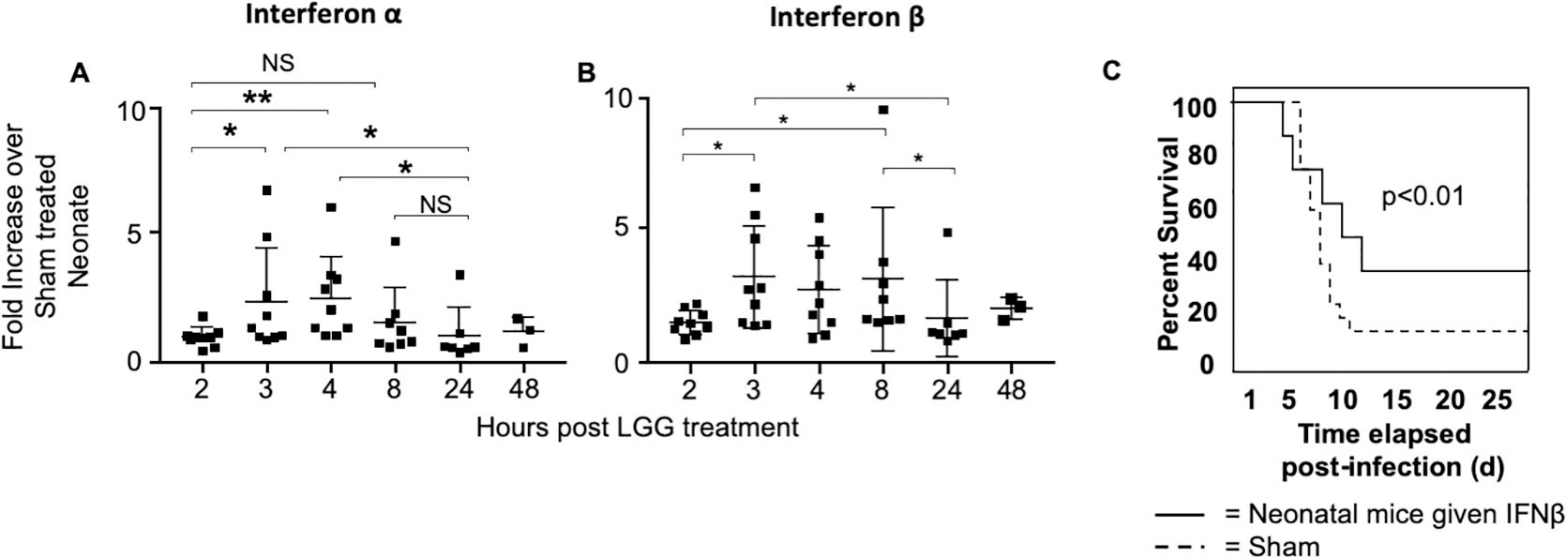
LGG promotes early transcription of Type I IFNs and survival of influenza virus-infected neonatal mice improves with IFNβ pretreatment. Mouse pups received 1 x10^6^ Colony Forming Units of LGG or sham intranasally on Day 1 of life and then harvested at various time points. Real time PCR was performed for **(A)** *IFNα4* and **(B)** *IFNβ1*. N = 3–9 per time point, 3 experiments. **(C)** Low dose IFNβ or sham treatment was given on Days of 1 and 2 of life. Animals were infected with influenza virus and tracked for survival. Kaplan-Meier survival statistics were completed for all experiments. (n = 15 per group, 3 experiments).

Based on this transcriptional analysis and LGG-induced interferon signaling, we hypothesized that LGG promoted an early burst of type I IFNs prior to infection which contributed to improved survival. To examine this, uninfected mice were given one dose of LGG, and lungs were harvested at different time points, from 2 hours to 48 hours post-LGG treatment. Real time PCR was performed to determine differences in transcription of *IFNα4* and *IFNβ1*. We found that there is a clear spike in transcription of these type I IFNs at 3 to 4 hours post-LGG, which diminishes by 24 hours (Fig 7A and 7B). These data provide evidence that LGG prompts production of type I IFNs prior to influenza infection, which modulates the type I IFN pathway and induces an antiviral state within the lung.

### Type I IFN pretreatment is sufficient to protect neonatal mice

Our transcriptional data indicated an unresponsiveness to influenza virus in neonates and LGG-induced upregulation of type I IFN genes, which suggested early induction of type I IFN could be mediating the protective effect of LGG. To answer this question, mice were treated with 1000 units of recombinant IFNβ intranasally before influenza virus infection on days 1 and 2 of life, and then infected on day 3 of life. With this pretreatment, mice had improved survival to 40% (p<0.01) (Fig 7C). This indicated that early type I IFN induction is sufficient to confer protection to neonatal mice.

### Neonatal protection by *Lactobacillus rhamnosus* is TLR dependent

**Fig 8:**
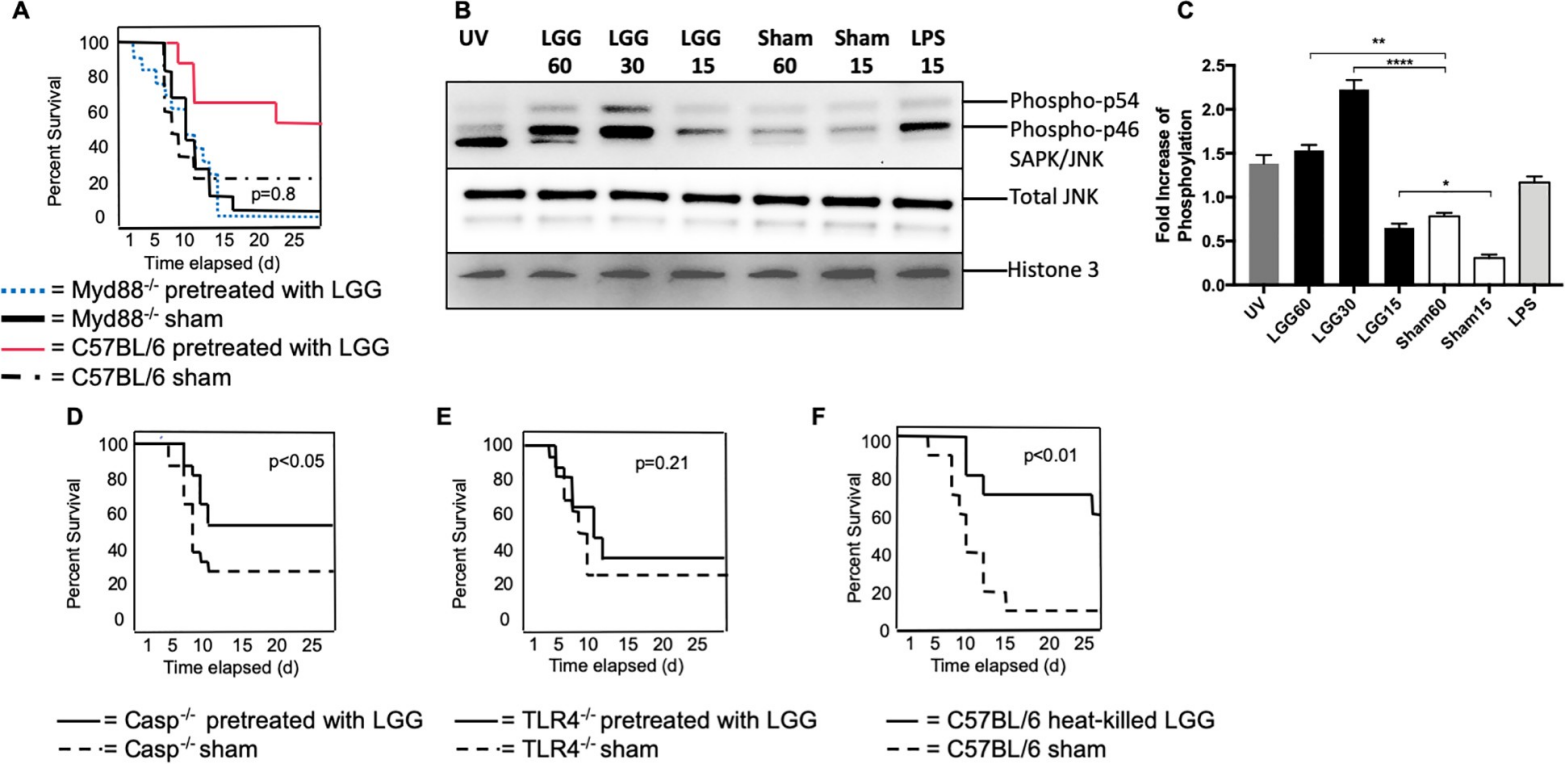
Heat-killed LGG provides equivalent protection and Toll like receptor signaling is critical for the recognition of *Lactobacillus*. **(A)** MyD88^-/-^ and C57BL/6 pups received 1 x10^6^ Colony Forming Units of LGG intranasally on Days 1 and 2 of life. On Day 3, pups were infected intranasally with PR-8 influenza, and survival was monitored. (p = 0.84, comparing the two MyD88^-/-^ groups) **(B)** A549 cells were treated with indicated conditions and proteins were isolated for a western blot of p-SAPK/JNK. (C) Quantification of phosphorylation of pSAPK/JNK compared to untreated control. A similar treatment and infection protocol was followed for **(D)** Casp1^-/-^ **(E)** TLR4^-/-^. N = 14–18 in each group from 4 independent experiments. **(F)** Mice were given 1 x10^8^ Colony Forming Units of heat-killed LGG intranasally on Days 1 and 2 of life, and then infected with influenza on the third day of life; survival was monitored. n = 17 per group from 4 independent experiments.

We next examined whether LGG was acting via pattern recognition receptor (PRR) pathways such as the Toll-like receptors (TLRs) [35] or the inflammasome [36]. To test TLR involvement, neonatal mice deficient for the adaptor protein myeloid differentiation factor (MyD88) were used, as MyD88^-/-^ animals cannot signal through the TLR pathway, with the exception of TLR3 [37]. We performed LGG pretreatment followed by influenza virus infection in MyD88^-/-^ and wild type neonatal mice and compared the survival of LGG- and sham-treated mice. We found that LGG protection in neonatal mice was abrogated when MyD88 was absent, which demonstrated that LGG recognition is MyD88 dependent (Fig 8A). In addition, neonatal mice were similarly sensitive to influenza virus infection without TLR signaling, which demonstrated that neonatal mortality is TLR signaling independent.

Activation of MyD88-dependent signaling requires downstream signaling cascades such as the MAPK pathway[38], whereby TLR activation leads to phosphorylation of tyrosine kinases such as stress-activated protein kinase/Jun-amino-terminal kinase (SAPK/JNK). As a surrogate for MAPK pathway activation, we examined phosphorylation levels of SAPK/JNK in a human alveolar epithelial cell line, A549. Cells were incubated with LGG and control media (MRS broth) for various times or treated with LPS or UV light, as positive controls. Cell lysates were used for immunoblotting for SAPK/pJNK. LGG-treated A549 cells demonstrated an increase in SAPK/JNK phosphorylation as early as 15 minutes compared to sham-treated cells, which indicated a rapid activation of downstream TLR pathways (Fig 8B and 8C).

To determine if another PRR, the inflammasome, was important for LGG protection, we tested LGG treatment in caspase-1 deficient mice (casp1^-/-^), which cannot signal through the inflammasome [39]. We found that influenza virus mortality of neonatal mice was independent of caspase-1 and that LGG continued to protect neonatal casp1^-/-^ mice (54% LGG versus 25% sham, p<0.05), thus excluding inflammasome involvement in both influenza virus-induced lethality and in the protective effect of LGG (Fig 8D). Finally, to determine the role of specific TLRs in the recognition of LGG, we used neonatal mice deficient in TLR4 (TLR4^-/-^), which is known to recognize bacterial components such as LPS [40], and has been shown to protect mice from influenza virus infection [41]. We performed LGG pretreatment and PR8 infection of TLR4^-/-^ and C57BL/6 mice and found that LGG protection was abrogated when TLR4 was absent (35% LGG versus 24% sham, p = 0.21; Fig 8E). While the canonical agonist for the TLR4 receptor is bacterial LPS, human and murine neonatal TLR responses differ from those of adults [42], with an increase in adult-like function during the first year of life [43]. There is a promiscuity in neonatal TLR in the ligands they respond to as well as intracellular crosstalk of their signaling [44]. Together, these studies highlight the protective effect of LGG is dependent upon a TLR4-MyD88 pathway in influenza virus-infected neonates.

### Heat-killed LGG is equally protective excluding colonization as a mechanism of LGG protection

When mice are given antibiotics prior to viral [9, 14] and bacterial [45] infection, the host is more susceptible, indicating that loss of commensal colonization on the mucosal surfaces leaves the host more vulnerable to infection. Therefore, we questioned if LGG colonization was required for protection of the neonatal animals. To test this, we examined whether giving the pups heat-killed bacteria would be as equally efficacious as live LGG. Previously, others have demonstrated a dose-response with heat-killed *Lactobacillus* [11]. Therefore, neonatal pups were given a larger dose of heat-killed LGG (1x10^8^CFU) on the first and second day of life and infected with influenza on the third day of life, similar to the live LGG treatment protocol. We found the pups were equally protected when given heat-killed LGG (p<0.01) (Fig 8F), indicating that colonization of the respiratory tract is not required for protection, but rather mucosal priming of the respiratory tract by a component of the LGG is important for protection.

## Discussion

Immediately following birth, neonates move from the sterile *in utero* environment to the external world, where they are exposed to many microorganisms, where tolerance is required to permit commensal colonization [46]. However, this tolerogenic environment can lead to a hyporesponsiveness to pathogenic organisms [47]. During this critical period, the neonate is unresponsive to respiratory viral infections. We demonstrate transcriptional paralysis in the neonatal mouse when infected with influenza virus. Twelve hours after infection, the neonatal mouse has a similar transcriptional profile to an uninfected mouse. The transcriptional response begins around 24-hours post infection, albeit with much less differentially expressed genes compared to LGG-treated neonates or adults. In addition, sham-treated influenza infected neonates have a different phenotypic response with a lack of early neutrophil recruitment at the site of infection. Potentially, the increase in morbidity and mortality associated with respiratory viral infections in neonates could be attributed to this kinetic shift in the antiviral response. To our knowledge, this is the first demonstration of an intranasal probiotic pretreatment improving survival after a respiratory viral infection in a neonatal animal model.

In contrast, there are profound transcriptional changes after influenza virus infection in adult mice [48] and humans [49]. Neonatal mice treated with *Lactobacillus rhamnosus* (LGG) prior to influenza virus infection can overcome this transcriptional unresponsiveness and are better equipped to combat the viral infection, as demonstrated by decreased early viral loads and improved survival. In addition, there are 32 genes down-regulated in LGG-treated influenza infected neonates, compared to sham-treated influenza treated neonates (Fig 6, S3 Table), and canonical pathway analysis revealed one pathway down-regulated in those animals which received LGG prior to influenza infection, Nitric oxide and Reactive Oxygen Species in macrophages. Virus-induced oxidative stress may contribute to several aspects of pathogenesis of influenza virus infection, including tissue damage, inflammatory response, and apoptosis[50]. The oxidative stress and damage induced during influenza virus infection is expected to be even greater in neonatal mice as it would exacerbate the oxidative stress known to be present in newborns [51]. Thus, newborns may be especially susceptible to the deleterious effects of oxidative stress. We speculate that reactive oxygen species, coupled with the neonatal inability to neutralize reactive oxygen species, could increase lung pathology in this vulnerable population.

Until recently, the lung was considered sterile in healthy individuals. Knowledge of the respiratory tract microbiota has changed dramatically over the past decade, with the advent of next generation sequencing. The composition of lung microbial communities has been associated with protection and plays a beneficial role in both allergic and infectious airway diseases [52, 53]. Recently, the importance of respiratory tract commensal colonization was demonstrated in a cohort of premature infants; colonization with *Lactobacillus* early after birth was associated with a decrease in bronchopulmonary dysplasia, a chronic lung disease of prematurity [54]. Similarly, oral probiotic treatment also reduces sepsis in term, healthy neonates [55]. Clinical epidemiologic observations further suggest that the immune effects of early-life microbial exposure persist into later life [13].

The role of the respiratory microbiome in health and disease is supported by studies using intranasal administration of *Lactobacillus* to ameliorate the symptoms of respiratory viral infection or allergic disease [10, 26, 27, 56]. In addition to the direct stimulation of pattern recognition receptors (PRRs) by commensal bacteria, metabolites of commensal bacteria can be protective during influenza infection and pulmonary allergic disease [57, 58]. Such signals could, however, be absent from neonates that have not been colonized yet with a lung microbiome. This quickly changes over the first few days as newborn mice already at 5 days of life have colonized their lung microbiome [59]. Although colonization may provide a steady anti-viral state in the lung, we show that inactivated LGG is sufficient to protect neonatal mice indicating that colonization is not required. Presumably, in the human setting, colonization would provide a long-term benefit as the effect of LGG administration diminishes with time.

PRRs that recognize gram positive bacteria and their components are considered important in *Lactobacillus*-mediated priming in the lung. The specific PRR utilized, however, might be different depending on the probiotic. For example, peptidoglycan from the *L*. *salivarius* strain Ls33 protected mice from chemical colitis in a nucleotide oligomerization domain (NOD) 2-dependent, MyD88-independent fashion [60], and *Lactobacillus plantarum* protection during Pneumonia Virus of Mice (PVM) infection was also found to be MyD88 independent [18]. However, others have found a direct link between MyD88 and *Lactobacillus* species [35]. Although originally described as receptors for bacteria and fungi, TLRs have become known as mediators of cytokine production in response to a variety of viruses and viral ligands. To test the role of TLRs in LGG recognition in neonatal animals, we undertook a series of survival experiments with mice devoid of the universal TLR adaptor protein MyD88 (MyD88^-/-^). In MyD88^-/-^ mice, LGG confers no protection, which implicates TLRs as the potential mechanism by which LGG provides neonatal mice protection. In contrast, Caspase-1 deficient neonatal mice, which have no inflammasome signaling, are still protected from influenza virus by LGG, excluding a role for the inflammasome. Further investigations into specific TLRs revealed that TLR4 is critical for the recognition of LGG. Interestingly, there are known differences in the function of TLR4 in the neonates[42, 61, 62]. Neonatal TLRs display promiscuity in their activating ligands[42] whereby a ligand is able to activate non-canonical receptors and induce varying effector functions[42]. LGG could modulate TLR4 and its downstream pathways[63–67]. LGG-treated intestinal epithelial cells have a lower inflammatory profile, especially lower levels of IL6 and IL8 [63], consistent with our findings. In addition, activation of TLR4 is important for induction of the Type I IFN responses and signaling [68].

Influenza virus has been shown to trigger type I IFN through recognition by TLR3, TLR7/8 and retinoic acid-inducible gene I-like receptors (RIG-I) in dendritic cells and alveolar epithelial cells [69]. IFNs are produced by virus-infected epithelial cells and induce an anti-viral state in cells receiving an IFN signal. Interferons, such as type I interferons (i.e., interferon-α, (IFN-α) and IFN-β) and type III interferons (i.e., IFN-λ2 and IFN-λ3 in mice; collectively called IFN-λ), are essential in antiviral defense [70–73]. Our *in vivo* experiments demonstrated that pretreatment with IFNβ protected neonatal mice during influenza virus infection, congruent with an infant mouse model of RSV infection [74]. Previous work demonstrated probiotic stimulated type I IFN production [75]; indeed, we found that LGG stimulated type I IFN transcription within hours of LGG treatment in murine neonates.

Innate response to influenza virus relies on pattern recognition molecules, like C-type lectins, RIG-I, TLRs and NOD-like receptors (NLRs) [76]. As compared to adults, the immune system of newborns is characterized by skewed TLR-mediated cytokine production, with impaired production of Th1-polarizing cytokines such as tumor necrosis factor and IL-12p70, but robust production of Th2/Th17-polarizing IL-6 [42, 77, 78]. TLR dimerization leads to the activation of nuclear factor-κB (NF-κB) and interferon-regulatory factors (IRFs), and these transcription factors in turn induce the production of proinflammatory cytokines and type I interferons (IFNs), respectively [79, 80]. There is conflicting data about which cell type is the source of type I IFNs during respiratory viral infection: epithelial cells, fibroblasts, plasmacytoid dendritic cells (pDCs), alveolar macrophages (AMs), and conventional DCs have all been shown to produce type I IFNs after virus exposure *in vitro* [81–85]. A primary reason for the conflicting data is that type I IFNs are made transiently and are difficult to detect *in vivo* [86]. As the immune system’s sentinel, pDCs differ drastically from conventional dendritic cells as they uniquely express TLR7 and TLR9. This exclusive TLR repertoire expression enables pDCs to be specialized in microbial nucleic acid sensing: TLR7 ligands include the poly-U ssRNA from influenza virus, whereas CpG dinucleotides, which are abundant in bacterial DNA, potently activate TLR9. pDCs serve as an important component of recognizing both viruses and bacteria and are an intriguing cell type which might be bridging the gap between probiotic treatment and viral infection. Potentially, there is early priming of pDCs by the inhaled probiotic; when the pup is exposed to virus, this important anti-viral cell type is already on the “front-line” and ready to produce large amounts of type I IFN. However, AMs have also been implicated in the production of type I IFNs and the subsequent recruitment of antiviral monocytes [86]. Although pDCs at the cellular level produce a hundred times more type I IFNs than other immune cells [87], they comprise a very small percentage of the cells at the site of influenza or RSV infection, particularly compared to the tissue resident macrophages, the AMs. Therefore, LGG may be acting on any of the above cell types to induce early type I IFN and promote an antiviral state that results in reduced viral loads early in infection.

Here, we report that neonatal mice exhibit an early lung transcriptional unresponsiveness at 12 hours post-infection, which begins to resolve by 24 hours post-infection, demonstrating a delay in the kinetics of the neonatal antiviral response. We show that a brief, two-day course of intranasal LGG prior to influenza virus infection protects neonatal mice from the mortality associated with infection. Transcriptional analysis reveals LGG pretreatment of neonates restores the lung transcriptional signature induced by infection to an adult-like profile. LGG-mediated protection is MyD88-dependent and specifically TLR4 dependent. Importantly, pups are equally protected when given heat-killed LGG, indicating that colonization of the respiratory tract is not required for protection, but primes the respiratory mucosa for a robust anti-influenza response. Future studies should determine the specific LGG components which stimulate type I IFN transcription and recognition by TLR4.

## Methods

### Mice, infections and Interferon β treatment

Eight-week old adult C57BL/6 mice were purchased from Charles River Laboratory to use as adult controls and for in-house breeding. MyD88^-/-^ (B6.129P2(SJL)-Myd88^tm1.1Defr^/J), Caspase 1^-/-^ (B6N.129S2-Casp1^tm1Flv^/J), and TLR4^-/-^ (B6(Cg)-Tlr4^tm1.2Karp^/J) mice were purchased from Jackson Laboratories. Experimental pups were obtained by timed mating in-house. The mice were housed under specific-pathogen-free conditions in an American Association for the Accreditation of Laboratory Animal Care-certified barrier facility at both the Drexel University College of Medicine Queen Lane Campus and New College Building animal facilities.

Neonatal mice at 3 days of age (weight ~3g) were infected intranasally (i.n.) with 0.12 TCID_50_ (0.04 TCID_50_ /g) of influenza virus H1N1 strain PR8 (A/Puerto Rico/8/34) in a 5 μl volume. Neonatal mice at 7 days of age (weight ~5g) were infected intranasally (i.n.) with 0.20 TCID_50_ (0.04 TCID_50_ /g) of influenza virus in a 7 μl volume. Adult 8-week old C57BL/6 mice (weight ~20g) were infected i.n. with a sublethal dose of 3 TCID_50_ in a 20 μl volume (0.15 TCID_50_ /g). The mice were anesthetized with inhaled isoflurane before intranasal inoculations. The pups were inspected daily for their activity level, respiratory rate, and the maternal interaction. If the pups were noted to be ignored/disregarded by the mother, had labored/fast breathing, weight loss, or lack of movement when handled, they were removed from the cage. However, majority of the pups did not exhibit signs of morbidity and were not found in the cage, assumed to be cannibalized by the mother.

Neonatal mice at 1 and 2 days of age were treated intranasally with 1,000 units of recombinant mouse Interferon β (Sigma-Aldrich, I9032). Recombinant IFN β was diluted in normal saline to 200 units per μl and 5 μl was administered intranasally under inhaled isoflurane anesthesia.

### Lactobacillus growth

LGG (ATCC 53103) was supplied by Valio Ltd (Helsinki, Finland) and cultured at 37°C for 16 hours in MRS broth (Lactobacillus broth according to De Man, Rogosa and Sharpe) (Becton, Dickinson & Co., Sparks, MD, USA). Mice were intranasally administered 5 μl LGG solution at a concentration of 1x10^6^ CFU once daily for two consecutive days. LGG was heat inactivated by heating to 95°C for 30 minutes and washed with PBS. Mice were intranasally administered 5 μl LGG solution at a concentration of 1x10^8^ CFU once daily for two consecutive days.

### Viral loads

At various time points post-infection, the lungs of the mice were harvested, weighed, and frozen at −80°C in TRIzol (Invitrogen). RNA was isolated by the Qiagen RNeasy kit (Qiagen). The isolated RNA was then used for cDNA synthesis using the High Capacity cDNA Reverse Transcription Kit (Applied Biosystems). Virus was measured by real-time PCR using influenza specific primers as previously described [88]. cDNA synthesis was performed using both a specific primer (5′-TCTAACCGAGGTCGAAACGTA-3′) and random hexamers. Real-time assays were performed in triplicate with 5 μl of cDNA, 12.5 μl of 2× TaqMan Universal PCR Master Mix (Applied Biosystems), 900 nM influenza A virus sense primer (5′-AAGACCAATCCTGTCACCTCTGA-3′), 900 nM influenza A virus antisense primer (5′-CAAAGCGTCTACGCTGCAGTCC-3′), and 200 nM influenza A virus probe (FAM-5′-TTTGTGTTCACGCTCACCGT-3′-TAMRA) [89]. All primers were specific for the influenza A virus matrix protein. Amplification and detection were performed using an Applied Biosystems Prism 7900HT sequence detection system with SDS 2.2.1 software (Applied Biosystems) at the following conditions: 2 min at 50°C and 10 min at 95°C, then 45 cycles of 15 s at 95°C and 1 min at 60°C. For viral load measurement, a standard curve was developed with serial 10-fold dilutions of stock PR8 with a known TCID_50_ concentration. Ct values were plotted against virus quantity in TCID_50_ per milliliter. This curve was used to convert the Ct values for viral loads to TCID_50_ equivalents. Virus RNA quantities in lungs were expressed as TCID_50_ equivalents/100 mg lung.

### Quantitative reverse transcription (RT)-PCR

At different points during infection, the right lobes of infected lungs were harvested and immediately homogenized in Tri Reagent (AB Applied Biosciences [AB]). Total RNA purification was carried out using the RiboPure kit (AB). Conversion into cDNA used the TaqMan RNA-to-CTT 2-step kit (AB). TaqMan quantitation of alpha 4 interferon (IFN-α4), beta 1 interferon (IFN-β1), gamma interferon (IFN-γ) IL-6, and GAPDH was carried out with inventoried primers in an AB 7900HT sequence detection system according to the manufacturer's instructions. For relative quantitation of the different mRNA species, all values were normalized to measured levels of GAPDH transcripts and expressed relative to values for uninfected WT mice using the comparative threshold cycle (*C*_*T*_) method (Applied Biosystems, Guide to performing relative quantitation of gene expression using real-time quantitative PCR, part number 4371095, rev. B).

### Isolation of pulmonary cells and Flow Cytometry

Pulmonary lymphocytes were isolated from individual mice by removing lungs and mincing into smaller pieces. The tissue was then digested for 2 h at 37°C with 3.0 mg/ml collagenase A and 0.15 μg/ml DNase I (Roche) in RPMI 1640 (Mediatech) containing 5% heat-inactivated FBS (Life Technologies), 2 mM l-glutamine, 100 IU/ml penicillin, 100 μg/ml streptomycin (Mediatech). The digested tissue was then passed through a 40-μm cell strainer (Falcon) and washed in the same media as above. Cells were counted using trypan blue exclusion with light microscopy.

Cells were co-stained with anti-mouse CD45 conjugated to PerCP Cy5.5 (Biolegend), CD11c conjugated to PE-Texas Red (Biolegend), CD11b conjugated to APC (Biolegend), Ly6G conjugated to Pacific Blue (Biolegend), MHCII conjugated to FITC (ThermoFisher), SiglecF conjugated to PE (Biolegend). All stains were completed on ice to prevent internalization. All absolute cell numbers are calculated per 100 mg of lung tissue. Cells were fixed in 1% paraformaldehyde (Fisher Scientific) before flow cytometric analysis. Data were collected on a FACS Fortessa using FACS Diva software (BD Biosciences). Analysis was performed using Flow Jo software (Tree Star).

### Histopathology

After lavage, the left lobe of the lung was inflated and fixed with 0.5 ml of 4% neutral-buffered formalin solution. Deparaffinized sections from fixed lungs were stained with hematoxylin and eosin (H&E). Lung infiltration was scored blindly by a board-certified pathologist. An intensity score in each of two categories was determined: peribronchiolotis (0 none to 5 severe) and alveolitis (0 none to 5 severe). A weighted intensity score was then determined based on the percentage of lung involvement.

### RNA isolation and Nanostring nCounter PanCancer Immune Profiling for Mouse

Total RNA was isolated from adult and neonatal lungs 12-hour post PR8 virus infection using RNeasy kit (Qiagen) and quantified using a ND-1000 sprectrophotometer (ThermoScientific). 50 ng of RNA was used per sample and diluted to 5 μL total using RNase free water for PanCancer Immune Profiling for Mouse (Nanostring). Samples were then processed according to nCounter Gene Expression Assay Protocol. In brief, 8 μL of master mix containing a hybridization buffer and Reporter CodeSet were added to each 5μL sample. 2 μL of Capture ProbeSet was then added to each sample, tubes were spun down and incubated overnight at 65°C in a pre-heated C1000 Touch Thermal Cycler (Bio-Rad). Samples were then quantified based on number of target probes using nCounter Digital Analyzer High Sensitivity setting and analyzed using nSolver software. Downstream analysis used exported count tables to identify differentially expressed genes applied the DESeq2 [90] [91]v 1.22.2) package, using counts of the NanoString house-keeping genes as “negative” control genes during estimation of size factors. In pairwise comparisons, Wald tests were used to measure statistical significance using an FDR-corrected p-value cut-off of 0.05. To measure non-additive interactions in gene expression with viral infection and probiotic treatment, likelihood ratio tests were performed, measuring the effect of including an infection:probiotic interaction term.

### Pathway analysis

Significantly upregulated and downregulated genes in whole lung lysate at 12 and 24 hours post influenza infection were determined based on a False Discovery Rate of <0.05 and greater than a 2-fold difference. Their fold-change values were uploaded into Ingenuity Pathway Analysis (IPA)(Qiagen). IPA analysis identified canonical pathways and upstream regulators predicted to be affected by the upregulated and downregulated genes. Blue indicates downregulated genes; orange indicates upregulated genes.

### Immunoblotting

For detection of pSAPK/JNK proteins, cells were seeded into a 6-well dish at 1x10^6^ cell per well and allow to rest overnight at 37°C in 5%CO2 incubator. LGG was grown as described previously. Cells were treated with LGG, Sham, LPS 100ng/ml, or UV light for the indicated amount of time. Cell monolayers were washed twice with PBS and whole cell lysates were obtained using RIPA buffer containing protease and phosphatase inhibitors (Cell Signaling Technology, 5872). Proteins were resolved in 5–20% gradient SDS-PAGE, then transferred to PVDF membranes for western blotting. Antibodies directed against the following proteins were used: Phospho-SAPK/JNK (Thr183/Tyr185) (Cell Signaling Technology, 4668), SAPK/JNK (Cell Signaling Technology, 9252) and Histone H3 (Cell Signaling Technology, 4499). Goat-anti-rabbit horseradish peroxidase conjugated secondary antibody was used for indirect detection of target proteins.

### Statistical analysis

Statistical analysis was performed using the Shapiro-Wilk W test for normality, Student's t-test and nonparametric Wilcoxon signed-rank test for paired and unpaired samples, log-rank test for survival curves. Analyses were performed with the JMP statistical analysis program (SAS, Cary, NC). P values<0.05 were considered to be statistically significant.

### Ethics statement

All experimental procedures and handling of mice were approved by the Drexel University College of Medicine Institutional Animal Care and Use Committee (IACUC), protocol number 20362, project number 1044999. All work was conducted in compliance with government regulations including the US Animal Welfare Act (Animal Welfare Assurance number A3222-01) and the Public Health Service Policy on Humane Care and Use of Laboratory Animals. The Animal Care and Use program at Drexel has received Full Accreditation from AAALAC International.

## Supporting information

S1 Table List of 194 differentially regulated genes in LGG-treated animals with log2 fold change and FDR <0.05. (TIFF)

S2 Table List of the 278 top upregulated genes in the LGG treated neonate group, 215 of which are shared with the adult. (TIFF)

S3 Table List of 32 genes that are downregulated in LGG-treated infected neonates compared to sham-infected neonates. (TIFF)

S1 Fig Flow cytometry gating strategy for immune-phenotyping of lung and airway immune cell infiltrates. (TIFF)

S2 Fig PCA of 12 and 24 hours post infection transcriptional analysis of uninfected, sham-treated influenza infected, LGG-treated influenza infected, LGG-treated uninfected neonates, and adults. (TIFF)
