# Supplementary figures and images for: Lung transcriptional unresponsiveness and loss of early influenza virus control in infected neonates is prevented by intranasal Lactobacillus rhamnosus GG
Source: PLoS Pathog. 2019 Oct 11;15(10):e1008072. doi: 10.1371/journal.ppat.1008072 (PMC6808501; doi:10.1371/journal.ppat.1008072)

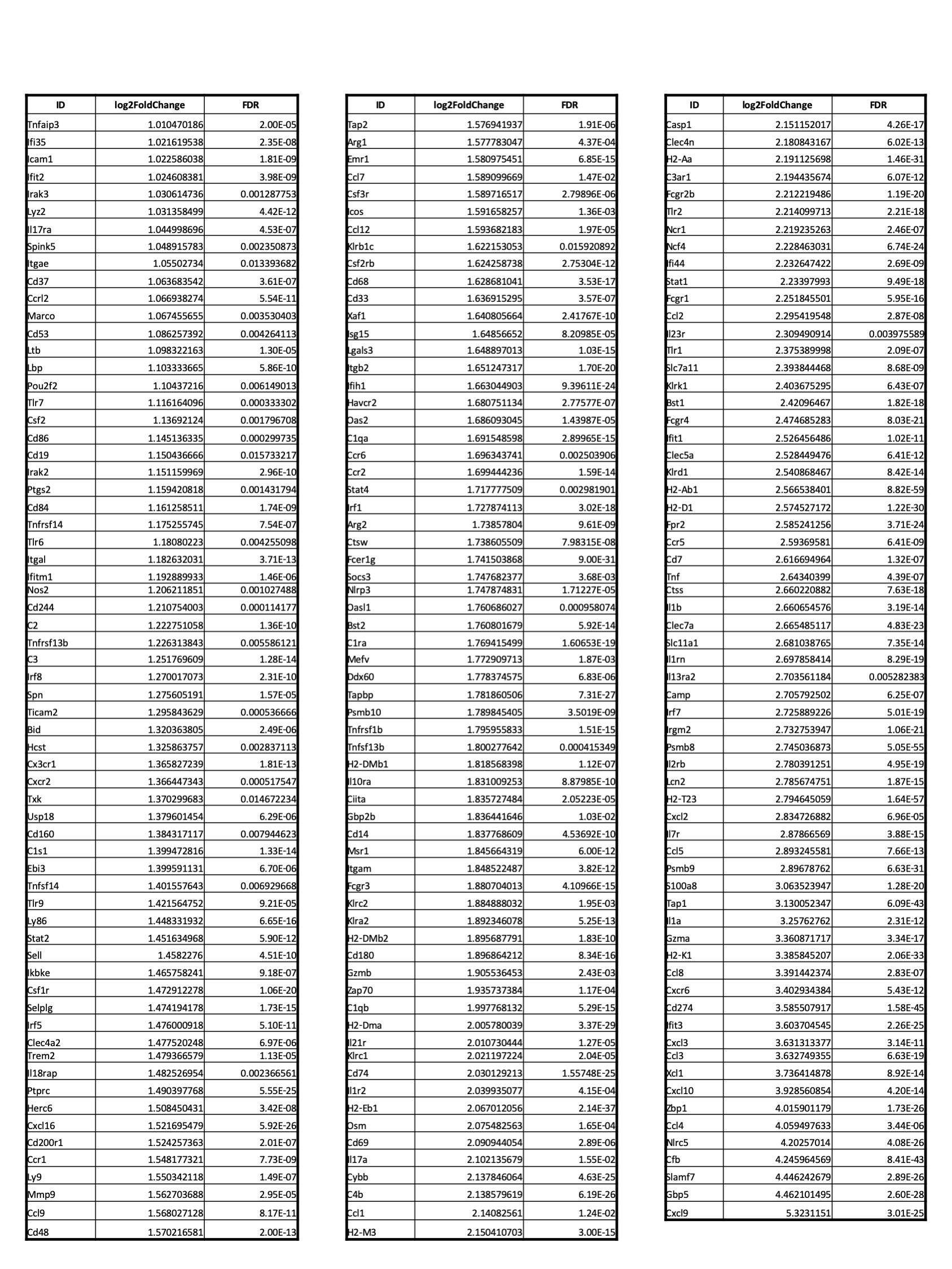

Supplement: S1 Table — (TIFF) [file ppat.1008072.s001.tiff]

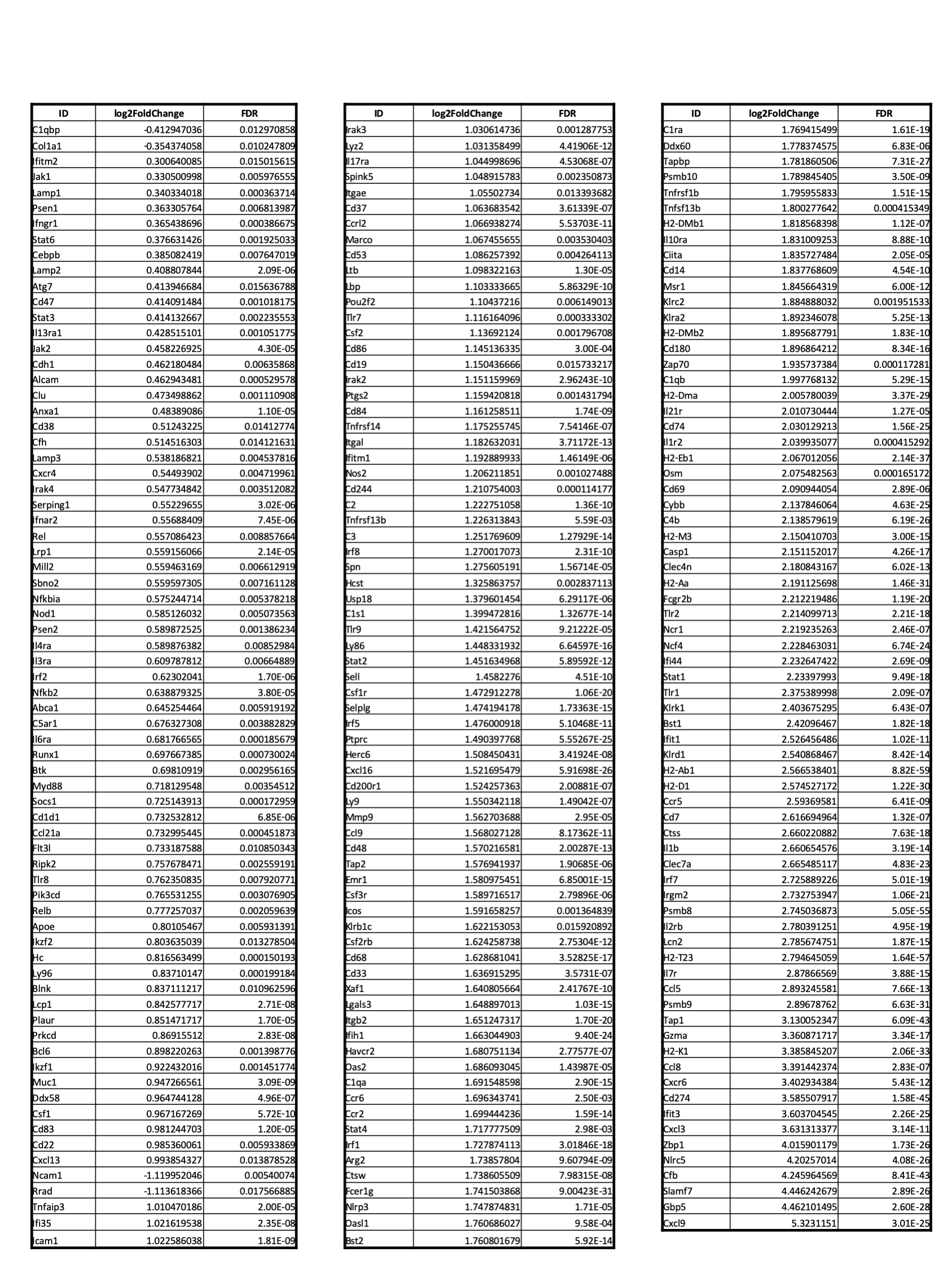

Supplement: S2 Table — (TIFF) [file ppat.1008072.s002.tiff]

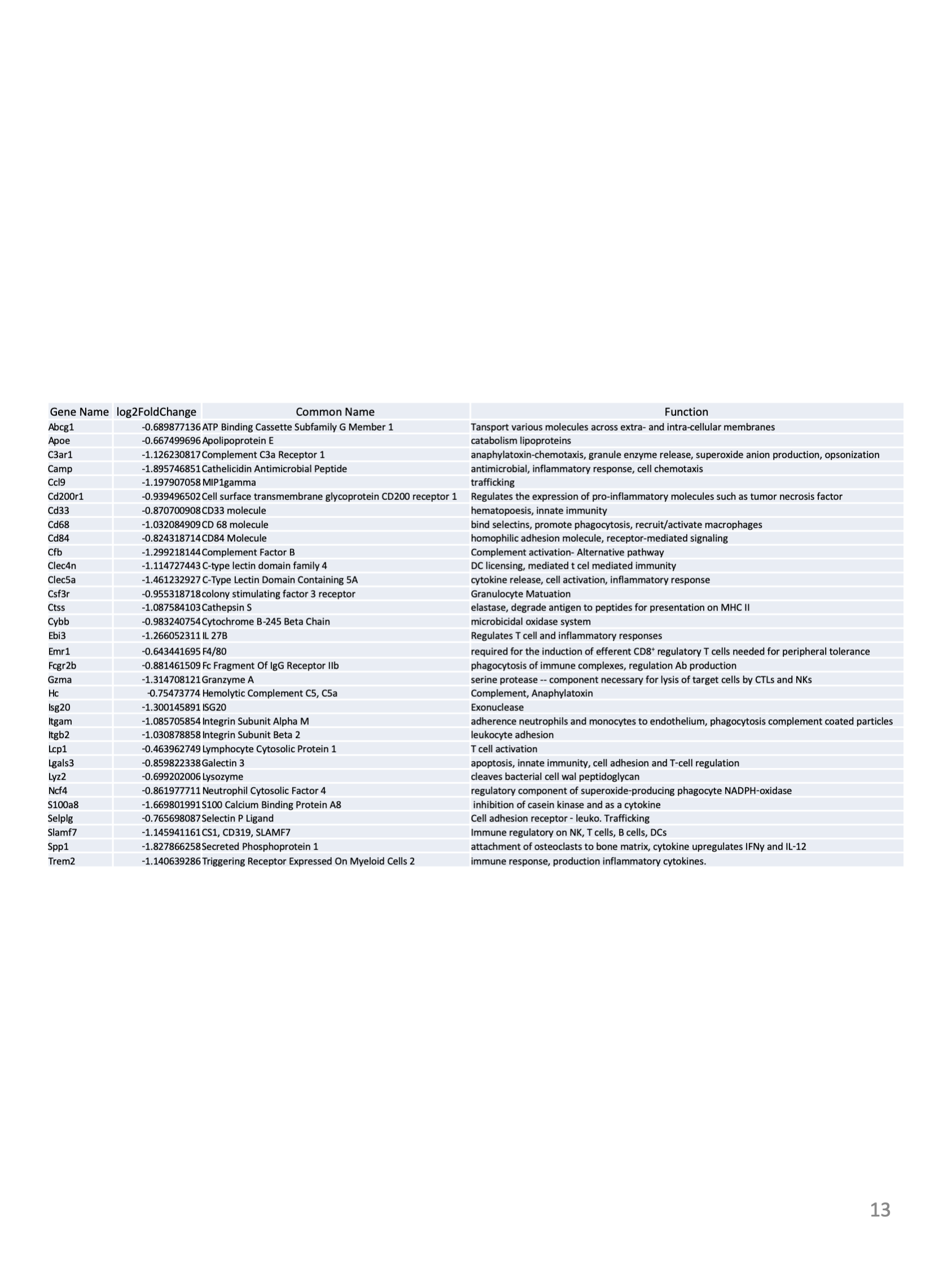

Supplement: S3 Table — (TIFF) [file ppat.1008072.s003.tiff]

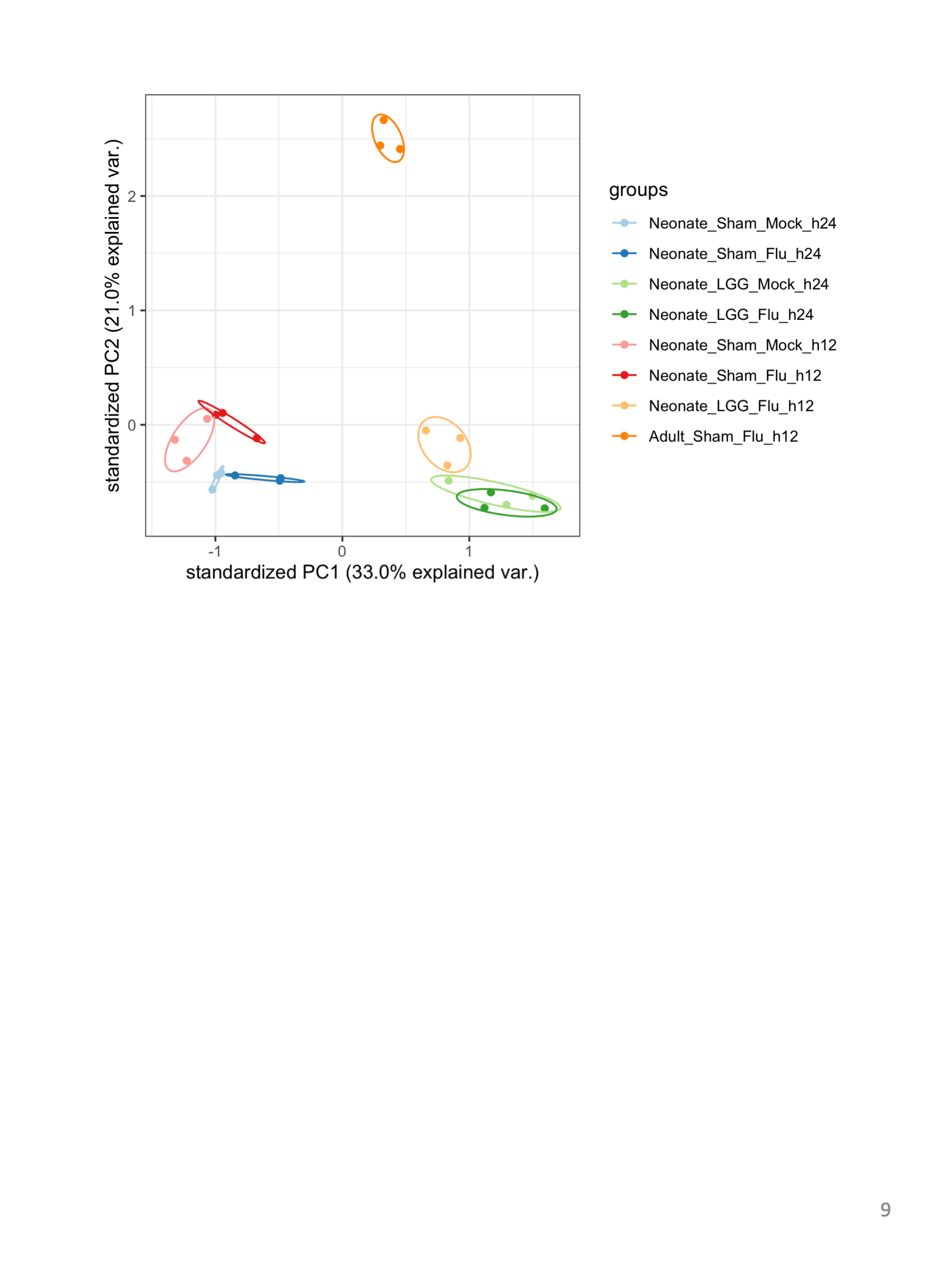

Supplement: S1 Fig — (TIFF) [file ppat.1008072.s004.tiff]

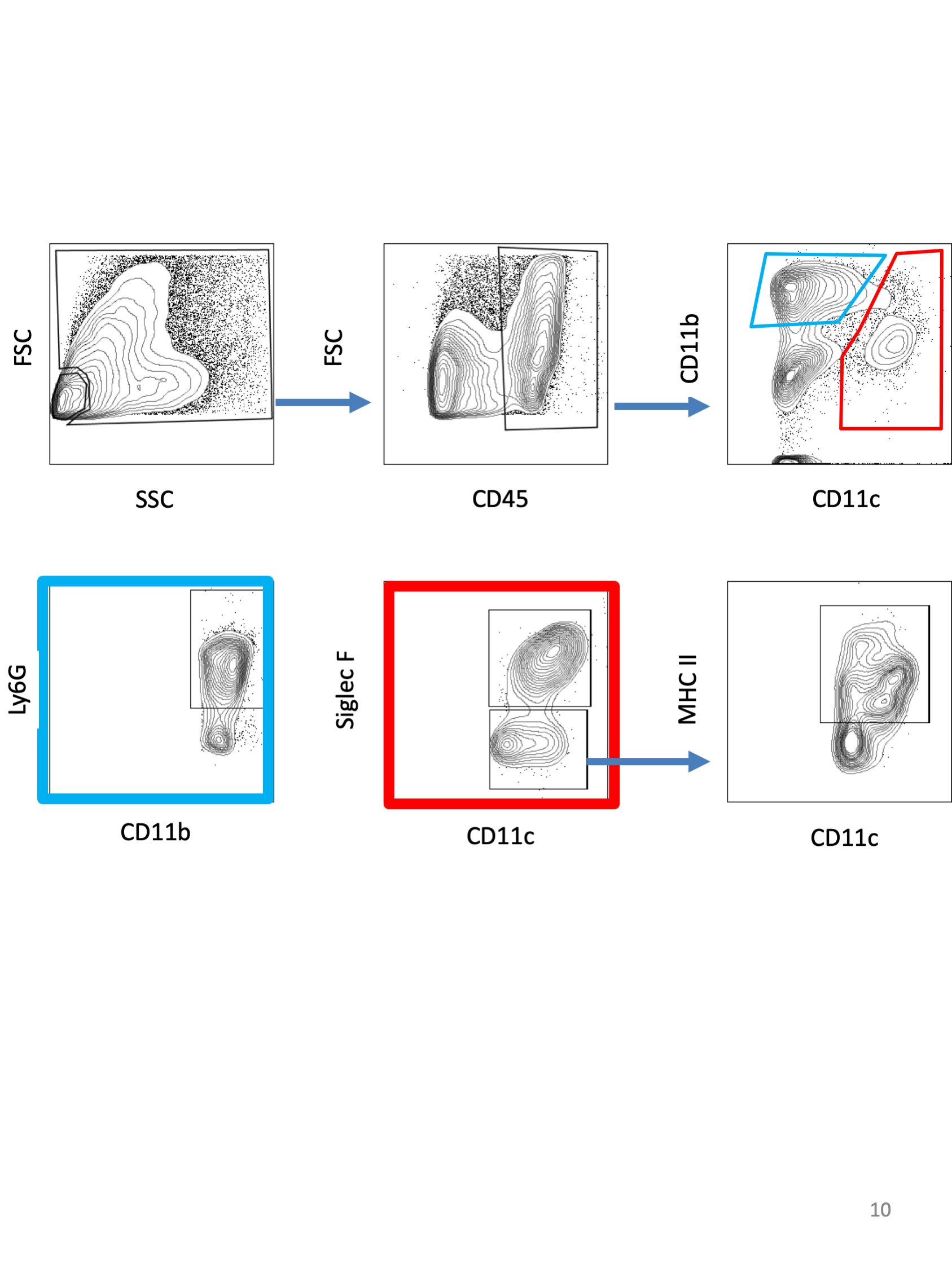

Supplement: S2 Fig — (TIFF) [file ppat.1008072.s005.tiff]
